# A multi-center nested case-control study on hospitalization costs and length of stay due to healthcare-associated infection

**DOI:** 10.1186/s13756-018-0386-1

**Published:** 2018-08-09

**Authors:** Yu Lü, Min Hong Cai, Jian Cheng, Kun Zou, Qian Xiang, Jia Yu Wu, Dao Qiong Wei, Zhong Hua Zhou, Hui Wang, Chen Wang, Jing Chen

**Affiliations:** Healthcare-associated Infection Management Office, Sichuan Academy of Medical Sciences and Sichuan People’s Hospital, Chengdu, 610072 Sichuan People’s Republic of China

**Keywords:** Healthcare-associated infections, Hospitalization costs, Length of stay, Multi-center, Nested case-control study

## Abstract

**Background:**

In 2018, the Chinese government demanded nationwide implementation of medical insurance payment methods based on Single-Disease Payment (SDP), but during the operation process the medical insurance system did not fully consider the extra economic burden caused by healthcare-associated infection (HAI). HAIs can prolong the length of stay and increase the hospitalization costs, but only a few studies have been conducted in Sichuan province, China. We evaluated the hospitalization costs and length of stay due to HAI in Sichuan province based on the prevalence survey, and provided data reference for China’s medical insurance reform.

**Methods:**

In the hospitals surveyed on the prevalence of HAI, a multi-center nested case-control study was performed by a paired method. The study period was from 6 September 2016 to 30 November 2016. Binary outcomes were tested using χ2 test, continuous outcomes were tested using Wilcoxon matched-pairs signed rank test, intra-group comparisons were tested using multiple linear regression analysis.

**Results:**

A total of 225 pairs/450 patients were selected in 51 hospitals, and 170 pairs/350 patients were successfully matched. The case fatality rate was 5.14% for the HAIs patients and 3.43% for non-HAs patients, there was no significant difference (χ^2^ = 0.627, *P* = 0.429); the median length of stay in patients with HAIs was 21 days, longer than that of patients with non-HAI 16 days, the median of the difference between matched-pairs was 5 days, the difference was statistically significant (*Z* = 4.896, *P* = 0.000). The median hospitalization costs of patients with HAI were €1732.83, higher than that of patients with non-HAI €1095.29, the median of the difference between matched-pairs were €431.34, the difference was statistically significant (*Z* = 6.413, *P* = 0.000). Multiple linear regression results showed that HAIs at different sites have caused different economic burdens, but in different economic regions, the difference was not statistically significant.

**Conclusions:**

In Sichuan, the hospitalization costs and length of stay caused by HAI should be given special attention in the current medical insurance reform. The proportion and scope of medical payment for patients with HAI at different sites should be different. Efforts need to be taken to incentivize reduction of HAI rates which will reduce hospitalization costs and length of stay.

## Background

In 2018, the Chinese government demanded nationwide implementation of medical insurance payment methods based on Single-Disease Payment (SDP) [1], but during the operation process the medical insurance system did not fully consider the extra economic burden caused by complications such as healthcare-associated infections (HAIs). After implementing SDP, most of the extra economic burden will be borne by the hospitals. This situation may lead to the risk that reimbursement cannot cover costs, so that hospitals have the motivation to reduce the quality of medical care, such as refusing patients with certain diseases to be admitted, reducing necessary treatments, or allowing patients to repeat hospital admissions [2, 3]. In order to make the reform successful, it is necessary to study the extra economic burden caused by HAI.

HAIs can bring a serious burden to patients [4], and the situation is in worse year by year [5]. HAIs can prolong the length of hospitalization stay, increase the hospitalization costs, and reduce the turnover rate of hospital beds [6, 7], which seriously affected the quality of medical care. There were many studies in this area, but less in Sichuan province, China. A total of 16.54 million hospitalizations were reported in Sichuan province in 2016 [8], and the prevalence rate of HAI was 2.30% [9]. It is important to understand and assess the hospitalization economic burden due to HAI in order to strengthen the management of HAI. The prevalence survey of HAI in Sichuan was designed by the HAIs quality control center established by Health and Family Planning Commission of Sichuan Province, using a unified questionnaire to complete within the prescribed time period. It has been conducted annually since 2011, and 6 rounds have been completed. Because of the support of the health administration department and the current prevalence survey had been included hospital grade review, the survey data in Sichuan became more accurate and large. This study was based on the prevalence survey, so that the accuracy and completeness of the data were reliable.

Since 2003, the outbreak of atypical pneumonia [severe acute respiratory syndromes (SARS)]has rapidly promoted a series of regulations and standards for HAI management in China, such as the Regulations of Healthcare Associated Infection Management and Accreditation Guideline of Control and Prevention of Healthcare Associated Infection in Hospital, etc. With the implementation of laws and regulations, the current basic measures for prevention and control of HAI in China were basically consistent with the general international standards [10]. The measures for prevention and control in our study were carried out in accordance with the requirements of the Action Plan for Prevention and Control of Healthcare Associated Infection in Sichuan Province (2012–2015) issued by the Health and Family Planning Commission of Sichuan Province in 2012. The contents of this action include departmental organization and management, education and training, surveillance, hospital layout and workflow, isolation, disinfection and sterilization, hand hygiene, occupational exposure protection, high-risk department management, intubation-associated infection management, disposable sterile medical supplies and disinfection equipment management, medical waste and wastewater management [11], and these measures were not significantly different from other provinces in China. The HAIs Quality Control Center of Sichuan Province organized quality control supervision once a year. Supervising team members conducted quality supervision on the implementation of measures for prevention and control of HAI. After the results of the inspection were reported to the Health and Family Planning Commission of Sichuan Province, they were notified and required to be rectified within the prescribed time.

## Methods

### Setting

This was a matched-pairs nested case-control study. All the subjects were followed up until they were discharged.

### Objects

Fifty one hospitals from 21 prefectures (municipality) were included in the prevalence survey of HAI in Sichuanin 2016.

### Selection of cases

The case was defined as those who had definite exposure to HAI during the hospitalization from 6 September 2016 to 30 November 2016. The HAI diagnostic criteria used in this work were issued by the Health and Family Planning Commission of China in 2001 [12]. A total of 10 cases were randomly selected from all patients with HAI in each of the hospital sunder the prevalence survey, and in hospitals with less than 10 HAI cases, all the cases were included.

Systematic sampling steps:

- Step 1: Encode all HAI cases obtained from the current prevalence survey;
- Step 2: Divide the total number of HAI cases by 10 to get the sampling interval;
- Step 3: Use the last few digits of a randomly drawn RMB currency to determine the first case. The selection principle of these digits was that the number to be taken was the maximum number but not greater than the sampling interval;
- Step 4: Select the second case by taking the number of first case plus the sampling interval; and so on, select all cases of HAI.

### Selection of controls

Controls were the non-HAI patients who met the matching criteria.

Controls were patients that did not yet acquire infection on the ‘matching day’ but that still can acquire infection later on during their stay. So controls were matched by department of discharge, age, sex, the total length of stay before infection, community-acquired infections, immune function, repeated hospitalization.

Matching criteria:

- ① Select patients in the same ward with HAI cases;
- ② In the present prevalence survey, the patients without HAI were selected;
- ③ It was necessary to eliminate the confounding bias of pre-infection time. So the total length of hospitalization stay before ‘matching day’ (Interval between the ‘matching day’ and the admission date) of the selected controls should be longer than or equal that of the HAI cases before infection (Interval between the infections date and admission date);
- ④ Select the patient with the same gender and in the same age group as the cases, and the age difference should be within 5 years as far as possible; The control of infant case aged <1 year should be the infant aged <1 year;
- ⑤ The following status of control need to be consistent with the case: whether there was community acquired infections at the time of admission, whether to be hospitalized for 2 times in a year, whether there were tumors, AIDS, malnutrition and other low immune function.
- ⑥ The classification score of the controls needed to be consistent with that of HAI cases. (Scoring method in the standard of nosocomial infections monitoring in China [13] Table 1).

**Table 1:** Classification score of disease conditions

Score	Classification basis
1	Routine observation was required and care and treatment were not required (including patients who need to be observed after surgery).
2	Stable condition, but preventive observation is needed, it is not necessary to strengthen care and treatment of patients, for example, some patients for whom myocarditis, myocardial infarction need to be excluded and overnight observation is needed after taking medicine.
3	Stable condition, but care and/or guardianship of patients need to be strengthened, such as coma patients or patients with chronic renal failure.
4	Unstable conditions, increased care and treatment are needed, and regular evaluation and adjustment of treatment programmes are needed, such as arrhythmia, diabetic ketoacidosis patients (but no coma, shock, DIC).
5	Unstable conditions, in a coma or shock state, cardiopulmonary resuscitation is needed or nursing care needs to be strengthened, and the care and treatment of patients need to be evaluated.

### Confounders

In confounding factors, we considered the hospital scalesand local economic development index -GDP. The areas were classified by GDP, the hospital scales were classified by the number of beds, and the assignment was shown in Table 2.

**Table 2:** Variable assignment

Variable	Value
Area (classified by GDP)	
Chengdu	1
Mianyang, Deyang, Yibin, Nanchong, Luzhou, Dazhou, Leshan, Liangshan	2
Panzhihua, Suining, Meishan, Guang’ an, Neijiang, Zigong	3
Ziyang, Guangyuan, Ya’ an,Bazhong, Aba, Ganzi	4
Number of hospital beds	
< 300	1
300 ≤ and < 500	2
500 ≤ and < 800	3
≥ 800	4

### Calculation of costs

Hospitalization costs: For each case and each control, the total bill for the entire hospital stay was obtained through the medical records. It was calculated as the sum of the cost of general medical service and general treatment operation, nursing costs, pathological diagnosis costs, laboratory diagnostic costs, imaging diagnostic costs, clinical diagnostic item costs, non-surgical clinical physiotherapy expenses, surgery treatment costs, rehabilitation costs, medicine costs, antibacterial drugs fees, Chinese herbal medicines fees, and the costs of disposable medical materials for examination, treatment or surgery.

### Statistical analysis

Binary outcomes were tested using χ^2^ test, continuous outcomes were tested using Wilcoxon matched-pairs signed rank test, intra-group comparisons were tested using multiple linear regression analysis, and the test level was α = 0.05. Analyses were stratified by the scale of hospital (number of hospital beds), local economic development (GDP) and the different sites of HAIs. All the analyses were conducted using software SPSS 23.0.

## Results

### Patient inclusion

A total of 225 pairs were enrolled in the study, in which 175 pairs were matched. Due to the different hospitalization time, 48 pairs and 96 patients were not matched successfully.

**Fig. 1:**
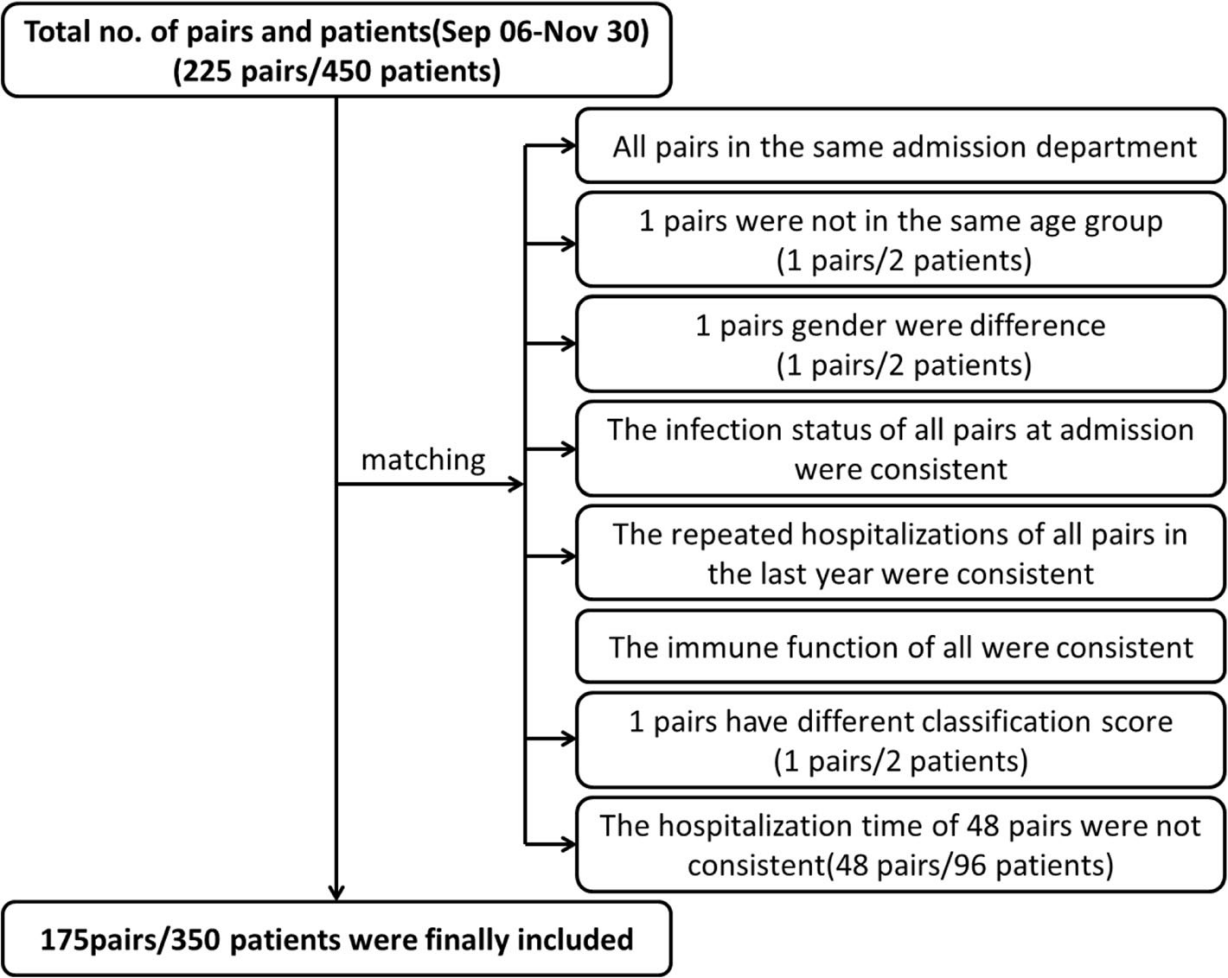
Flow chart of Patients inclusion. In the matching process, 48 pairs were excluded because they did not meet the total length of hospitalization stay before ‘matching day’. One pair was excluded because of not meeting the age factor. One pair was excluded because of not meeting the gender factors. One pair was excluded because of not meeting the classification score of disease conditions. After matching, the final 175 pairs/350 patients were included

Data were cleared up by matching, and pairs that did not meet the matching criteria were excluded, and finally 175 pairs were included in the analysis. See Fig. 1 for details.

### Characteristics of patients

The study included a total of 175 pairs of cases and controls in 51 hospitals, as showed in Table 3 and Table 4.

**Table 3:** Pairs characteristics

Variable	Number of Hospitals (*n* = 51)	Number of Pairs (*n* = 175)	Ratio of composition
Classification of GDP			
1	4	19	10.86%
2	23	71	40.57%
3	11	37	21.14%
4	13	48	27.43%
Classification of the number of hospital beds			
1	19	29	16.57%
2	11	47	26.86%
3	13	49	28.00%
4	8	50	28.57%

**Table 4:** Patient characteristics: cases and controls

Variable	Cases(*n* = 175)	Controls(*n* = 175)
Mean age ± SD	58.15 ± 20.56	57.76 ± 20.09
Classification score of disease conditions		
1	40(22.9%)	40(22.9%)
2	63(36.0%)	63(36.0%)
3	42(24.0%)	42(24.0%)
4	26(14.9%)	26(14.9%)
5	4(2.3%)	4(2.3%)
Gender		
Male	99(56.6%)	99(56.6%)
Female	76(43.4%)	76(43.4%)
Community infections	39(22.3%)	39(22.3%)
Repeated hospitalization	41(23.4%)	41(23.4%)
Low immune function	29(16.6%)	29(16.6%)

### Overall analysis

The case fatality rate of HAI group was 5.14% (9/175), the case fatality rate of non-HAI group was 3.43%(6/175), there was no significant difference (χ^2^ = 0.627, *P* = 0.429). The median length of hospitalization stay of HAI patients was 21 days, longer than that of non-HAI patients 16 days, the median of the difference between matched-pairs was 5 days, the difference was statistically significant (*Z* = 4.896, *P* = 0.000). The median hospitalization costs of HAI patients were €1732.83, longer than that of non-HAI patients €1095.29, the median of the difference between matched-pairs were €431.34, the difference was statistically significant (*Z* = 6.413, *P* = 0.000).

### Stratified analysis

No matter the number of hospital beds, the length of additional hospitalization stay was higher in patients with HAI than in patients without HAI and the difference was statistically significant. Except for GDP level 2, in other economic regions the differences in length of stay were statistically significant.

The results at different sites of HAI showed that there were statistically significant differences in length of stay for upper respiratory infections, superficial incision infections, urinary infections, and lower respiratory infections. See Table 5 for details.

**Table 5:** Comparison of length of stay between the matched pairs (Days)

Variable	Number of Pairs (*n* = 175)	Difference of length of stay in pairs Median (range)	Statistics (*Z*)	*P*
Classification of the number of hospital beds				
1	29	4.0(−8~ 107)	3.136	0.002
2	47	4.0(−30~ 52)	2.131	0.033
3	49	6.0(−56~ 134)	2.716	0.005
4	50	6.0(−94~ 52)	2.045	0.041
Classification of GDP				
1	19	5.0(−23~ 69)	2.439	0.015
2	71	3.0(−94~ 134)	1.712	0.087
3	37	4.0(−30~ 88)	2.106	0.035
4	48	7.0(−39~ 107)	3.847	0.000
HAIs sites				
Upper respiratory	23	3.0(−21~ 14)	− 2.236	0.026
Blood related	5	6.0(−22~ 20)	−0.135	0.893
Superficial incision	20	10.0(−3~ 107)	−3.680	0.000
Organ lacunar	2	4.5(3~ 6)	−1.342	0.180
Deep incision	6	12.0(−10~ 52)	− 1.802	0.072
Intra-abdominal tissue	1	5.0(5~ 5)	/	/
Urinary	23	6.0(−13~ 88)	−2.480	0.013
Skin soft-tissue	12	1.0(−14~ 32)	0.157	0.875
Lower respiratory	76	5.0(−94~ 134)	−2.439	0.015
Gastrointestinal	6	1.0(−22~ 54)	−0.674	0.500
Other	1	11.0(11~ 11)	/	/

Regardless of the number of hospital beds, or the economic conditions in the area, hospitalization costs caused by HAI were higher in patients with HAI, and the differences were statistically significant. The results at different sites of HAI showed that there were statistically significant differences in hospitalization costs for blood related infections, superficial incision infections, deep incision infections, urinary infections, lower respiratory infections, and gastrointestinal infections. See Table 6 for details.

**Table 6:** Comparison of hospitalization costs between the matched pairs (€)

Variable	Number of Pairs (*n* = 175)	Difference of costs in pairs Median (range)	Statistics (*Z*)	*P*
Classification of the number of hospital beds				
1	29	133.06(− 986.46~ 7891.43)	2.983	0.003
2	47	194.23(− 1163.39~ 8198.11)	2.106	0.035
3	49	748.94(− 2815.11~ 25,447.11)	4.491	0.000
4	50	572.45(− 6589.03~ 31,153.30)	2.106	0.035
Classification of GDP				
1	19	1362.03(− 270.13~ 25,447.11)	3.662	0.000
2	71	477.72(− 1632.29~ 31,153.30)	4.111	0.000
3	37	230.68(−6589.03~ 6712.01)	2.195	0.028
4	48	253.22(−2815.11~ 9800.81)	2.913	0.004
HAIs sites				
Upper respiratory	23	69.08(−986.46~ 761.57)	−1.186	0.236
Blood related	5	1163.39(390.33~ 1578.86)	−2.023	0.043
Superficial incision	20	574.77(− 1117.95~ 25,447.11)	−3.136	0.002
Organ lacunar	2	685.30(283.52~ 1087.09)	−1.342	0.180
Deep incision	6	1406.22(399.98~ 3431.36)	−2.201	0.028
Intra-abdominal tissue	1	2086.92(2086.92~ 2086.92)	/	/
Urinary	23	330.14(−6589.03~ 6712.01)	−2.201	0.043
Skin soft-tissue	12	39.06(−789.99~ 4542.83)	−0.078	0.937
Lower respiratory	76	993.88(− 6064.87~ 31,153.30)	−5.486	0.000
Gastrointestinal	6	51.63(−12.32~ 5168.36)	− 1.992	0.046
Other	1	335.81(335.81~ 335.81)	/	/

### Multiple linear regression analysis

**Fig. 2:**
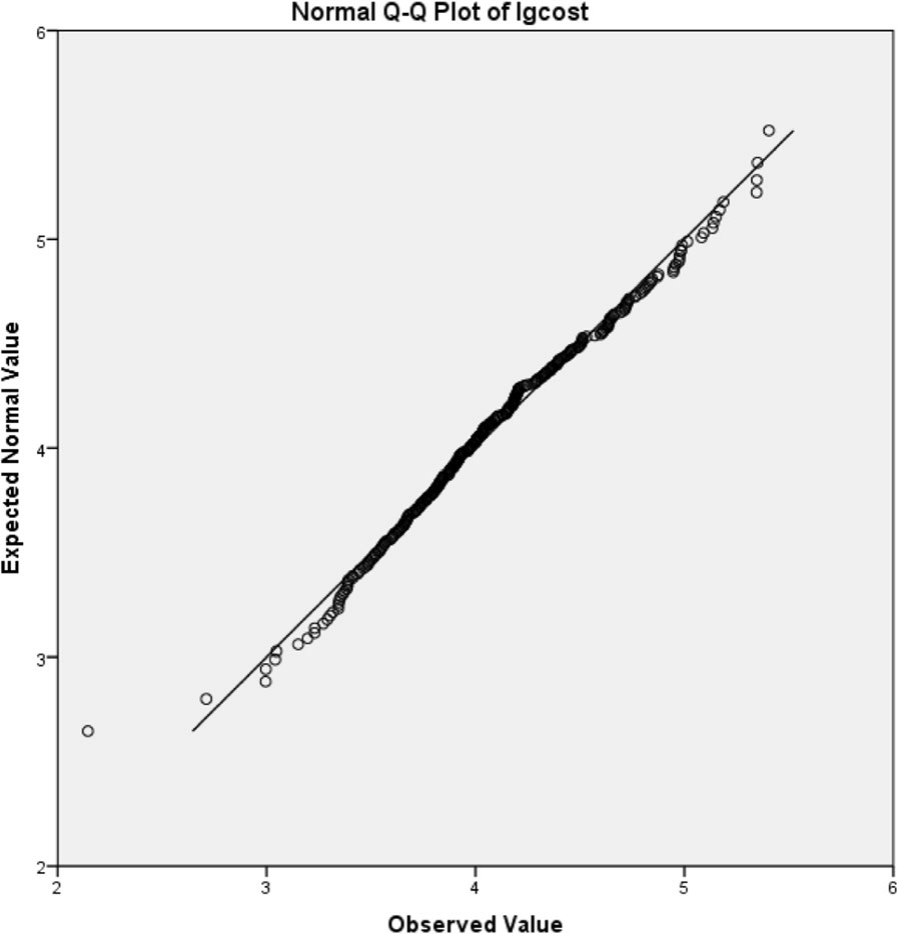
Q-Q diagram after logarithmic conversion of hospitalization costs. Most of the scatter distribution was on a diagonal line, suggesting that the data was normally distributed after conversion

The hospitalization costs and length of stay might have significant differences in different sites of HAIs. Multiple linear regression methods were used to analyze whether the differences were statistically significant. The cost was skewed data, so logarithmic conversion was performed on it. After conversion the Skewness was 0.164, Std. Error of Skewness was 0.184, and Kurtosis was 0.362, Std. Error of Kurtosis was 0.366, which satisfied the normal distribution. The Q-Q chart was showed in Fig. 2.

The F value of the ANOVA test for linear regression was 4.995, *P* = 0.000. The regression results showed that compared with upper respiratory infections, patients with these infections have higher extra hospitalization costs, such as blood related infections, superficial incision infections, organ lacunar infections, deep incision infections, urinary infections and skin soft-tissue infections, and the differences were statistically significant. See the Table 7 for details.

**Table 7:** Covariate and equation parameters for linear regression of hospitalization costs

Variable	Unstandardized Coefficients	Std. Error	Standardized Coefficients	Statistics (*t*)	*P*
Constant	3.820	.120		31.944	.000
HAIs sites					
Blood related	.762	.208	.265	3.661	.000
Superficial incision	.482	.128	.320	3.760	.000
Organ lacunar	.673	.308	.149	2.184	.030
Deep incision	.774	.195	.294	3.961	.000
Intra-abdominal tissue	.695	.435	.109	1.595	.113
Urinary	.511	.124	.360	4.120	.000
Skin soft-tissue	.422	.151	.222	2.789	.006
Lower respiratory	.606	.104	.625	5.820	.000
Gastrointestinal	.060	.190	.023	.315	.753
Other	−.049	.429	−.008	−.114	.909
Classification of GDP					
1	.088	.124	.057	.710	.479
2	−.052	.081	−.053	−.649	.517
3	−.076	.096	−.064	−.784	.434
Classification of the number of hospital beds					
1	−.241	.102	−.184	−2.367	.019
2	−.162	.090	−.149	−1.795	.075
3	.013	.097	.012	.138	.890

**Fig. 3:**
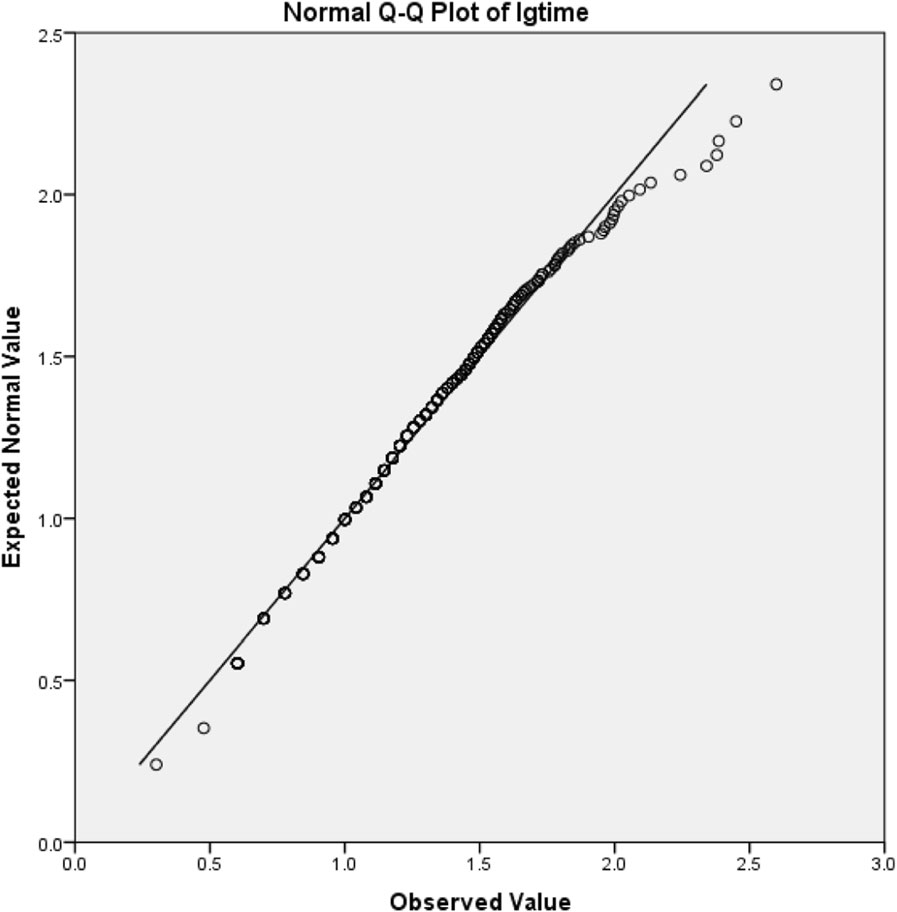
Q-Q diagram after logarithmic conversion of length of stay. Most of the scatter distribution was on a diagonal line, suggesting that the data was normally distributed after conversion

The length of stay was skewed data, so logarithmic conversion was performed on it. After conversion the Skewness was 0.479, Std. Error of Skewness was 0.186; and Kurtosis was 0.544, Std. Error of Kurtosis was 0.369, which satisfied the normal distribution. The Q-Q chart was showed in Fig. 3.

The F value of the ANOVA test for linear regression was 3.083, *P* = 0.000. The results showed that compared with upper respiratory infections, patients with these infections have longer extra hospitalization stays, such as blood related infections, superficial incision infections, deep incision infections, urinary infections, and lower respiratory infections, and the differences were statistically significant; the differences may not be statistically significant due to the small sample size, such as organ lacunar infections and intra-abdominal tissue infections. See the Table 8 for details.

**Table 8:** Covariate and equation parameters for linear regression of length of stay

Variable	Unstandardized Coefficients	Std. Error	Standardized Coefficients	Statistics (*t*)	*P*
Constant	1.269	.087		14.533	.000
HAIs sites					
Blood related	.315	.152	.162	2.077	.039
Superficial incision	.305	.093	.299	3.261	.001
Organ lacunar	.070	.224	.023	.314	.754
Deep incision	.579	.142	.326	4.066	.000
Intra-abdominal tissue	.184	.317	.043	.580	.563
Urinary	.288	.090	.301	3.198	.002
Skin soft-tissue	.135	.117	.097	1.153	.251
Lower respiratory	.211	.076	.319	2.769	.006
Gastrointestinal	.041	.138	.023	.298	.766
Other	.278	.312	.065	.891	.374
Classification of GDP					
1	−.028	.090	−.027	−.314	.754
2	−.094	.059	−.140	−1.586	.115
3	−.083	.070	−.104	−1.181	.239
Classification of the number of hospital beds					
1	−.120	.074	−.136	−1.620	.107
2	−.196	.067	−.262	−2.934	.004
3	.038	.070	.052	.535	.593

## Discussion

Our study was a prospective nested case-control study, using a paired method to exclude the effects of confounding factors. The results showed that HAI increased the length of stay and hospitalization costs, with an average of 5 days (increased by 31.25%), and an increase of inpatient costs of €431.34 (increased by 39.38%). We did not classify pathogens, and previous studies had shown that multi-drug resistant nosocomial infections could worsen the financial burden of patients [14–16]. A propensity matched case control study from Singapore, showed that the median per day costs of laboratory tests and antibiotics of patients with HAI were 1.5–2 times higher than that of patients without gram negative bacilli (GNB) infection [17]. A retrospective study from Spain, showed that the total hospital costs of patients with resistant and multidrug resistant *Pseudomonas aeruginosa* infection were 1.4 times and 1.7 times higher than those of patients with non-resistant *P.aeruginosa* infectio [18].

Although in the regions with different GDP levels, the relative number of extra economic burdens caused by HAI was quite different, the regression analysis results in our study showed that the difference was not statistically significant. In the previous studies, the extra economic burden caused by HAI varied greatly, such as Wu Anhua’s review suggests that the economic costs caused by HAI were related to the size of the survey hospital and the economic level of the hospital’s location [19]. but such differences still need to be further elaborated by more rigorous epidemiological methods. Our study found statistically significant differences only between hospitals of different sizes.

Because HAIs were considered to be preventable, the additional costs associated with HAI were avoidable, the SDP currently being tried by China in 2018 could stimulate hospitals to strengthen the management of HAI. However, our study found that the difference in extra economic burden caused by HAI at different sites was statistically significant. In the implementation of SDP, this situation should be regarded, and set different reimbursement ranges and proportions for HAI at different sites. For example, the Centers for Medicare and Medicaid Services (CMS) policy in the United States had done a good job, it covered 3 types of common infections: (1) selected surgical site infections, (2) vascular catheter-associated infections, and, (3) catheter-associated urinary infections [20]. In China, the management of HAI is not involved in the current medical insurance system. Incentives for HAI management are usually based on quality control reviews of clinical departments or medical staff [21], it is difficult to guarantee the effectiveness. However, it is worth noting that government leaders have also realized that HAI management can be motivated through better allocation of resources [22]. So the economic evaluation data in our study can provide reference for government decision-making in the future.

It was significant to match the hospitalization stay before ‘matching day’. Previous studies have shown that if the study did not consider the “risk time” before the infections occurred, it would result in a “time bias”, 2 times over estimation [23]. The study by Barnett AG reported that the additional hospitalization stay fell from 11.2 days to 1.4 days after adjusting the length of hospitalization stay before HAI [24]. Our study avoided the “time bias” by matching the period before the infections occurred between matched-pairs.

The data quality of our study was warranted because it was designed and completed within the framework of the prevalence survey, but simultaneously the sample size was limited and only 51 hospitals in 21 municipalities were investigated. Larger multi-center studies should be conducted to validate our findings.

### Limitations

The limitations of our study: the proportion of inpatient medical expense reimbursement was not considered, and the personal economic burden, the hospital economic burden and social burden caused by HAI were not differentiated.

## Conclusion

Our research shows that HAI significantly increase the length of stay and hospitalization costs, suggesting that efforts need to be taken to incentivize reduction of HAI rates which will reduce hospitalization costs and length of stay. Furthermore, the differences in extra hospitalization costs and length of stay caused by HAI at different sites were statistically significant. Thus, we suggest that when China focuses on the nationwide implementation of SDP policy reforms, in addition to paying more attention to the hospitalization costs and length of stay caused by HAI, the proportion and scope of medical payment for patients with HAI at different sites should be different.

## References

[1] Office of the Sichuan Provincial People's Government. Notice on Printing and Distributing the Implementation Plan for Further Deepening the Reform of Payment Methods for Basic Medical Insurance. Available from:http://www.sc.gov.cn/10462/12771/2018/1/8/10442346.shtml(2018). Accessed 26 July 2018.

[2] Di X (2018). The development of specific disease payment and its key issues of management. Chinese Health Resources.

[3] Zhi-jian LIU (2017). Study on the Effect of New Health Payment Scheme on Medical Costs. D. University of Science and Technology of China.

[4] Allegranzi B, Bagheri NS, Combescure C (2011). Burden of endemic health-care-associated infection in developing countries: systematic review and meta-analysis. Lancet.

[5] Guo C, Liu YH, Tian DS (2012). Statistical research on direct economic loss due to nosocomial infections. Chinese Journal of Nosocomiology.

[6] Vrijens F, Hulstaert F, Sande SVD (2010). Hospital-acquired, laboratory-confirmed bloodstream infections: linking national surveillance data to clinical and financial hospital data to estimate increased length of stay and healthcare costs. J Hosp Infect.

[7] Gabriel L, Beriot-Mathiot A (2014). Hospitalization stay and costs attributable to Clostridium difficile infection: a critical review. J Hosp Infect.

[8] Health and Family Planning Commission of Sichuan Province. Statistical communique on the development of health and family planning in Sichuan province in 2016.[EB/OL]. 2017–03-30. Available from: http://www.scwst.gov.cn/xx/tjxx/tjnj/201703/t20170330_13407.html. Accessed 26 July 2018.

[9] Sichuan Provincial Healthcare-associated Infections Quality Control Center. Report of 2016 Cross-sectional survey of healthcare-associated infection in Sichuan province. Sichuan Nosocomial Infection Control Network, 2017–06-26. Available from: http://www.scnicn.com:8081/zhuzhan/middlenews/20170626/812.shtml. Accessed 26 July 2018.

[10] Liuyi LI. New technique and progress of prevention and control of healthcare-associated infection. West China Medicine. 2018;33(3):240–43.

[11] Health and Family Planning Commission of Sichuan Province. Action Plan for Prevention and Control of Healthcare Associated Infection in Sichuan Province (2012–2015). [Z]. 2013–02-01. Available from: http://www.scnicn.com:8081/zcfl/836.jhtml. Accessed 26 July 2018.

[12] National Health and Family Planning Commission of the People's Republic of China. Notice on Issuing Diagnostic Criteria for Nosocomial Infection (Trial). [Z].2001–11-07. Available from: http://www.moh.gov.cn/yzygj/s3593/200804/e19e4448378643a09913ccf2a055c79d.shtml. Accessed 26 July 2018.

[13] Ministry of Health P.R. China (2009). WS/T 312–2009.Standard for nosocomial infection surveillance.

[14] Judd WR, Ratliff PD, Hickson RP (2016). Clinical and economic impact of meropenem resistance in Pseudomonas aeruginosa-infected patients. Am J Infect Control.

[15] Marta R, Pietro C, Roser T (2016). Cost Attributable to Nosocomial Bacteremia. Analysis According to Microorganism and Antimicrobial Sensitivity in a University Hospital in Barcelona. Plos One.

[16] Stewardson AJ, Allignol A, Beyersmann J, et al. The health and economic burden of bloodstream infections caused by antimicrobial-susceptible and non-susceptible Enterobacteriaceae and *Staphylococcus aureus* in European hospitals, 2010 and 2011: a multicentre retrospective cohort study. Euro surveillance. 2016;21(33):1–12. 10.2807/1560-7917.ES.2016.21.33.30319 PMC4998424 27562950

[17] Vasudevan A, Memon BI, Mukhopadhyay A (2015). The costs of nosocomial resistant gram negative intensive care unit infections among patients with the systemic inflammatory response syndrome- a propensity matched case control study. Antimicrobial Resistance and Infection Control.

[18] Morales E, Cots F, Sala M (2012). Hospital costs of nosocomial multi-drug resistant Pseudomonas aeruginosa acquisition. BMC Health Serv Res.

[19] Wu AH (2016). Economicevaluation onthe costs of healthcareassociated infection. Chinese Journal of Infection Control.

[20] Stone PW, Glied SA, Mcnair PD (2010). CMS changes in reimbursement for HAIs: setting a research agenda. Med Care.

[21] Zhang XJ, Qiu TF (2012). Application of incentive mechanism in control of nosocomial infections. Chinese Journal of Nosocomiology.

[22] Qiang FU, Guo YH (2013). National strategy for nosocomial infection control in new era. Chinese Journal of Nosocomiology.

[23] Schulgen G, Kropec A, Kappstein I (2000). Estimation of extra hospitalization stay attributable to nosocomial infections: heterogeneity and timing of events. J Clin Epidemiol.

[24] Barnett AG, Beyersmann J, Allignol A (2011). The time-dependent bias and its effect on extra length of stay due to nosocomial infection. Value in Health the Journal of the International Society for Pharmacoeconomics & Outcomes Research.

